# A validation study of potential prognostic DNA methylation biomarkers in patients with acute myeloid leukemia using a custom DNA methylation sequencing panel

**DOI:** 10.1186/s13148-022-01242-6

**Published:** 2022-02-11

**Authors:** Šárka Šestáková, Ela Cerovská, Cyril Šálek, Dávid Kundrát, Ivana Ježíšková, Adam Folta, Jiří Mayer, Zdeněk Ráčil, Petr Cetkovský, Hana Remešová

**Affiliations:** 1 Department of Genomics, Institute of Hematology and Blood Transfusion, U Nemocnice 1, 128 20 Prague 2, Czech Republic; 2 Institute of Clinical and Experimental Hematology, 1st Faculty of Medicine, Charles University, Prague, Czech Republic; 3 Faculty of Science, Charles University, Prague, Czech Republic; 4 Department of Internal Medicine-Hematology and Oncology, University Hospital Brno, Brno, Czech Republic

**Keywords:** DNA methylation, AML, Prognosis, Validation

## Abstract

**Background:**

Multiple studies have reported the prognostic impact of DNA methylation changes in acute myeloid leukemia (AML). However, these epigenetic markers have not been thoroughly validated and therefore are still not considered in clinical practice. Hence, we aimed to independently verify results of selected studies describing the relationship between DNA methylation of specific genes and their prognostic potential in predicting overall survival (OS) and event-free survival (EFS).

**Results:**

Fourteen studies (published 2011–2019) comprising of 27 genes were subjected to validation by a custom NGS-based sequencing panel in 178 newly diagnosed non-M3 AML patients treated by 3 + 7 induction regimen. The results were considered as successfully validated, if both the log-rank test and multivariate Cox regression analysis had a *p*-value ≤ 0.05. The predictive role of DNA methylation was confirmed for three studies comprising of four genes: *CEBPA* (OS: *p* = 0.02; EFS: *p* = 0.03), *PBX3* (EFS: *p* = 0.01), *LZTS2* (OS: *p* = 0.05; EFS: *p* = 0.0003), and *NR6A1* (OS: *p* = 0.004; EFS: *p* = 0.0003). For all of these genes, higher methylation was an indicator of longer survival. Concurrent higher methylation of both *LZTS2* and *NR6A1* was highly significant for survival in cytogenetically normal (CN) AML group (OS: *p* < 0.0001; EFS: *p* < 0.0001) as well as for the whole AML cohort (OS: *p* = 0.01; EFS < 0.0001). In contrast, for two studies reporting the poor prognostic effect of higher *GPX3* and *DLX4* methylation, we found the exact opposite, again linking higher *GPX3* (OS: *p* = 0.006; EFS: *p* < 0.0001) and *DLX4* (OS: *p* = 0.03; EFS = 0.03) methylation to a favorable treatment outcome. Individual gene significance levels refer to the outcomes of multivariate Cox regression analysis.

**Conclusions:**

Out of twenty-seven genes subjected to DNA methylation validation, a prognostic role was observed for six genes. Therefore, independent validation studies are necessary to reveal truly prognostic DNA methylation changes and to enable the introduction of these promising epigenetic markers into clinical practice.

**Supplementary Information:**

The online version contains supplementary material available at 10.1186/s13148-022-01242-6.

## Introduction

Acute myeloid leukemia (AML) is a hematopoietic malignancy characterized by a complex interplay of aberrations at different levels of the genome (i.e., genetic, epigenetic, transcriptomic, and proteomic) [1–3]. This complexity is faithfully reflected by AML heterogeneity in terms of pathogenesis and prognosis. In clinical practice, only properly introduced and validated genetic lesions altogether with cytogenetics are considered into treatment decision making [4]. This still applies despite growing evidence that some other markers, such as epigenetic factors, may add valuable information about the predicted course of the disease in individual AML patients [3]. DNA methylation is one of the longest-studied epigenetic mechanisms and is stable and relatively easy to measure [5, 6]. Therefore, its status can be readily harnessed as a clinically relevant stratifier. Indeed, there are an increasing number of articles assessing the influence of DNA methylation on AML prognosis—reviewed in [7]. These studies interrogate one, a few or multiple loci depending on the methodology used. Typically, as a result of such research, authors define gene(s) that may serve as new biomarkers to improve risk stratification in AML patients. The main weakness is that such works are usually not validated by other researchers and hence there is not sufficient validation of these potential biomarkers for them to be introduced into clinical practice. Therefore, we designed a comprehensive NGS-based DNA methylation panel comprising of genes previously published as having an impact on AML prognosis. For validation purposes, we selected fourteen studies published between years 2011 and 2019 [8–21] covering 27 genes (Additional file 1: Table S1). We chose works targeting only one or a few loci at once (averaged 2 loci per publication, range 1 to 7), because lower numbers of biomarkers would be more feasible for introduction into a laboratory routine practice. The list of the selected studies and their basic characterization is summarized in Table 1. The aim of this work was to make an independent verification of results published by other researchers to narrow down the list of actually prognostically relevant genes that may allow more precise AML stratification in the future.

**Table 1:** Studies subjected to DNA methylation validation

Publication	Studied region/gene	Sample type	Methylation detection method	Clinical significance	Notes	Test cohort (n)	Validation cohort (n)
Lin et al*.* [13]	*CEBPA* distal promoter	BM	Bisulfite sequencing, quantitative MassArray	Higher methylation was associated with longer OS in AML with normal karyotype without *CEBPA*^mut^ and *NPM1*^mut^, and in AML excluding favorable karyotype, *CEBPA*^mut^ and *NPM1*^mut^	Methylation of the *CEBPA* distal promoter inversely correlated with *CEBPA* expression	193 de novo AML, prognostic significance in CN-AML without *CEBPA*^mut^ and *NPM1*^mut^ (*n* = 25) and in AML excluding favorable karyotype, *CEBPA*^mut^ and *NPM1*^mut^ (*n* = 59)	None
Hájková et al*.* [8]	Promoters of tumor suppressor genes (*CDKN2B, ESR1, MYOD1, CALCA, SOCS1, CDH1*)	PB or BM MNC	MethyLight PCR	Hypermethylation of *SOCS1* promoter associated with better outcome. Patients with smaller number of hypermethylated genes (*p* = 0.012) or with lower levels of cumulative DNA methylation value computed from methylation levels of all studied regions have worse OS. and EFS	Studied negative impact of *HOX* genes and tumor suppressors promoters hypomethylation caused by *DNMT3A* mutations	79 diagnostic AML excluding favorable karyotype	None
Treppendahl et al*. *[18]	*VTRNA2-1* promoter	BM	pyrosequencing	Patients with hypermethylation (≥ 10% or > 38%) had poorer survival	Methylation was inversely correlated with expression	101 diagnostic AML	None
Hájková et al*.* [10]	*PBX3* (TAF1 binding site)	PB or BM MNC	NGS, pyrosequencing	Lower methylation correlated with higher expression of *PBX3* that was associated with higher incidence of relapse	Newly discovered hypomethylation pattern specific to *CBFB-MYH11* fusion with corresponding gene overexpression	123 diagnostic AML, prognostic significance in 40 AML that underwent standard curative therapy and did not die during the first induction	None
Jost et al*.* [11]	Promoter region of *DNMT3A*	PB	TCGA data, pyrosequencing (validation)	Hypermethylation (> 10%) associated with shorter EFS and OS in TCGA data, but not validated on authors' cohort of patients	Higher methylation in the region was mostly observed in patients without *DNMT3A*^mut^ and was associated with moderate downregulation of *DNMT3A* transcription	194 diagnostic AML of TCGA study, prognostic significance after excluding DNMT3^mut^ AML	88 diagnostic AML, prognostic significance not validated
Marcucci et al*.* [15]	DMRs in promoters of seven genes (*CD34, RHOC, SCRN1, F2RL1, FAM92A1, MIR155HG,* and *VWA8*)	BM	NGS: MethylCap enriched by MBD2, RRBS (validation), MassArray (validation)	High DMRs methylation associated with lower expression linked to higher CR rate and longer survival in CN-AML. Patients with lower weighted summary score of expression levels had higher disease-free survival and OS	*FLT3*-ITD and *DNMT3A* mutations associated with low methylation at DMRs, *NPM1* and *IDH* mutations associated with higher methylation at DMRs	134 CN-AML	four independent CN-AML patient sets (* n* = 355)
Božić et al*.* [21]	One CpG in *C1R* gene	PB	TCGA data, pyrosequencing (validation)	Higher methylation (> 27%) associated with longer OS	Only moderate association of DNA methylation and expression of *C1R*	194 diagnostic AML of TCGA study	two independent datatasets—62 CN-AML and 84 AML
Zhou et al*.* [19]	*GPX3* promoter	BM MNC	qMSP	non-M3 AML patients with *GPX3* methylation showed shorter OS	*GPX3* methylation does not correlate with expression	181 de novo AML, clinical significance in 104 non-M3 AML	none
Zhou et al*.* [20]	*DLX4*	BM MNC	qMSP	Patients with methylated *DLX4* presented lower CR rate and shorter OS	*DLX4* methylation was negatively associated with the expression of shorter *DLX4* isoform	133 de novo AML	None
Guo et al*.* [9]	*SFRP1* and *SFRP2* promoter regions	BM	qMSP	Higher methylation associated with shorter OS	Higher *SFRP1* methylation associated with N/K-RAS mutations. Higher *SFRP*s methylation in older patients (≥ 50 years)	139 de novo non-M3 AML	None
Li et al*.* [12]	*NKD2* promoter	BM MNC	qMSP	Higher methylation correlated with lower expression of *NKD2* which was associated with shorter OS in CN-AML	The role of DNA methylation in silencing of *NKD2* expression was confirmed in THP1 leukemic cell line	101 diagnostic AML, clinical significance proved in 42 CN-AML	Two independent datatasets—162 CN-AML and 78 CN-AML
Liu et al*.* [14]	*RASSF1A* promoter	BM	qMSP	Hypermethylation connected with decreased OS and EFS	Hypermethylation of *RASSF1A* associated with *ASXL1* mutations and decreased mRNA levels	226 diagnostic non-M3 AML	None
Qu et al*.* [16]	CGI shores of *LZTS2* and *NR6A1*	PB or BM	CHARMcox, pyrosequencing (validation), TCGA data (validation)	Hypomethylation in either of the two regions associated with worse OS	Studied on CN - AML patients	72 CN-AML in discovery cohort + 65 CN-AML in model-building cohort	65 CN-AML + 93 CN-AML from TCGA study
Šestáková et al*.* [17]	*GZMB* enhancer	PB	pyrosequencing	Hypermethylation associated with inferior OS between high and low methylation groups)	Concurrent presence of both *DNMT3A*^mut^ and *IDH1/2*^mut^ partially cancel out the opposite influence of these aberrations on DNA methylation resulting in a mixed methylation and hydroxymethylation profiles	104 diagnostic AML	None

BM, bone marrow; CGI, CpG island; CN-AML, cytogenetically normal AML; CR, complete remission; DMR, differentially methylated region; EFS, event-free survival; MNC, mononuclear cells; OS, overall survival; PB, peripheral blood

MassArray, Mass spectrometry analysis of cleaved fragments of chosen regions amplified by PCR; TCGA data, data from The Cancer Genome Atlas Research Network 2013 AML study [36]; qMSP, quantitative methylation-specific polymerase chain reaction; CHARMcox, Comprehensive High-throughput Array-based Relative Methylation Analysis combined with Cox proportional Hazards Model; RRBS, Reduced representation bisulfite sequencing

## Results

**Fig. 1:**
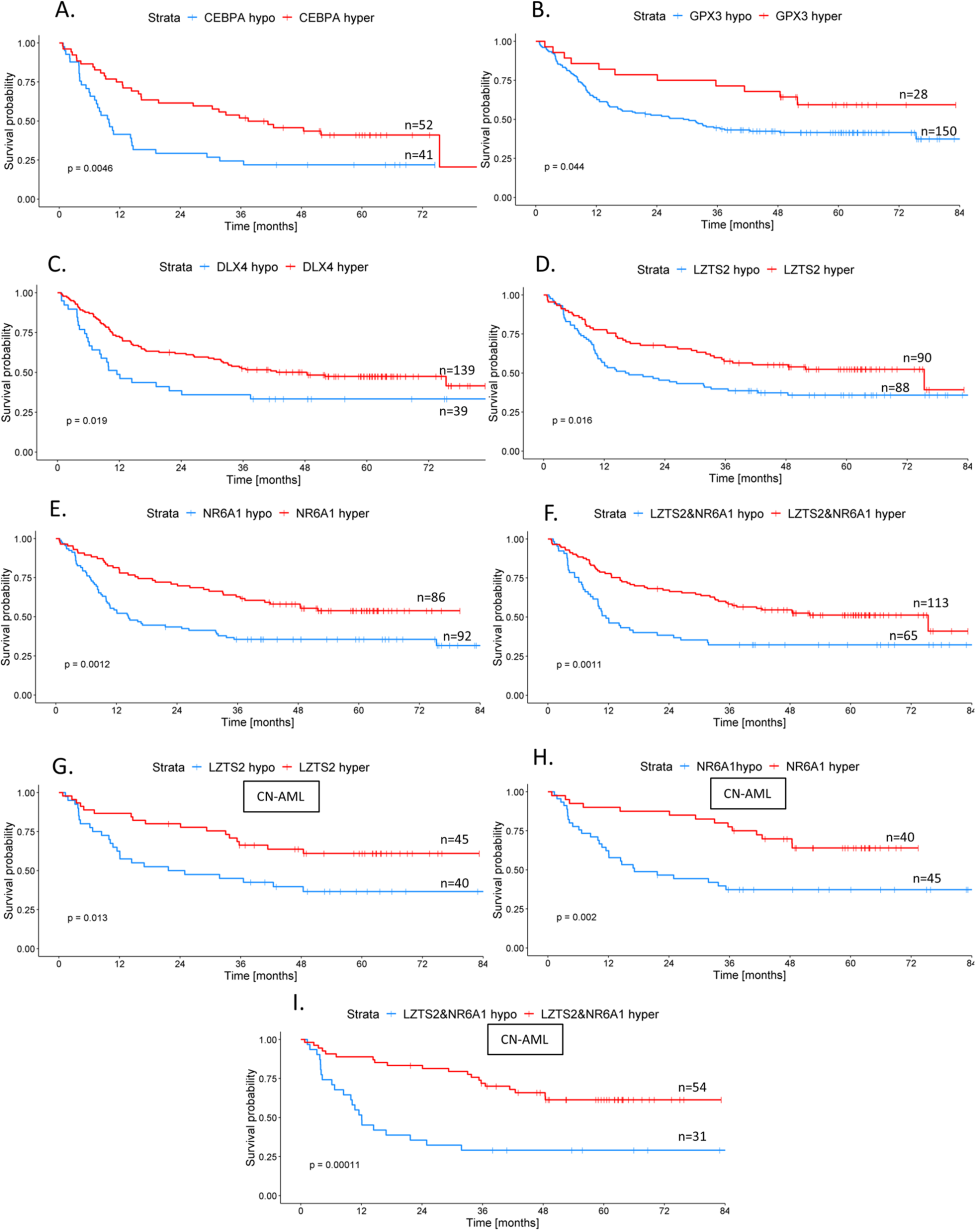
Kaplan–Meier (KM) curves for overall survival (OS): **A** *CEBPA* methylation KM curves in AML subgroup excluding favorable cytogenetics and without *CEBPA* and *NPM1* mutations (*n* = 83). **B** *GPX3* methylation KM curves in the whole non-M3 AML cohort (*n* = 178). **C** *DLX4* methylation KM curves in the whole non-M3 AML cohort (*n* = 178). **D** *LZTS2* methylation KM curves in the whole non-M3 AML cohort (*n* = 178). **E** *NR6A1* methylation KM curves in the whole non-M3 AML cohort (*n* = 178). **F** *LZTS2*&*NR6A1* methylation KM curves in the whole non-M3 AML cohort (*n* = 178). **G** *LZTS2* methylation KM curves in the CN-AML subgroup (*n* = 85). **H** *NR6A1* methylation KM curves in the CN-AML subgroup (*n* = 85). **I**   *LZTS2*&*NR6A1* methylation KM curves in the CN-AML subgroup (*n* = 85). CN-AML = cytogenetically normal AML, hypo = hypomethylated, hyper = hypermethylated, Strata—stratified by a variable

**Fig. 2:**
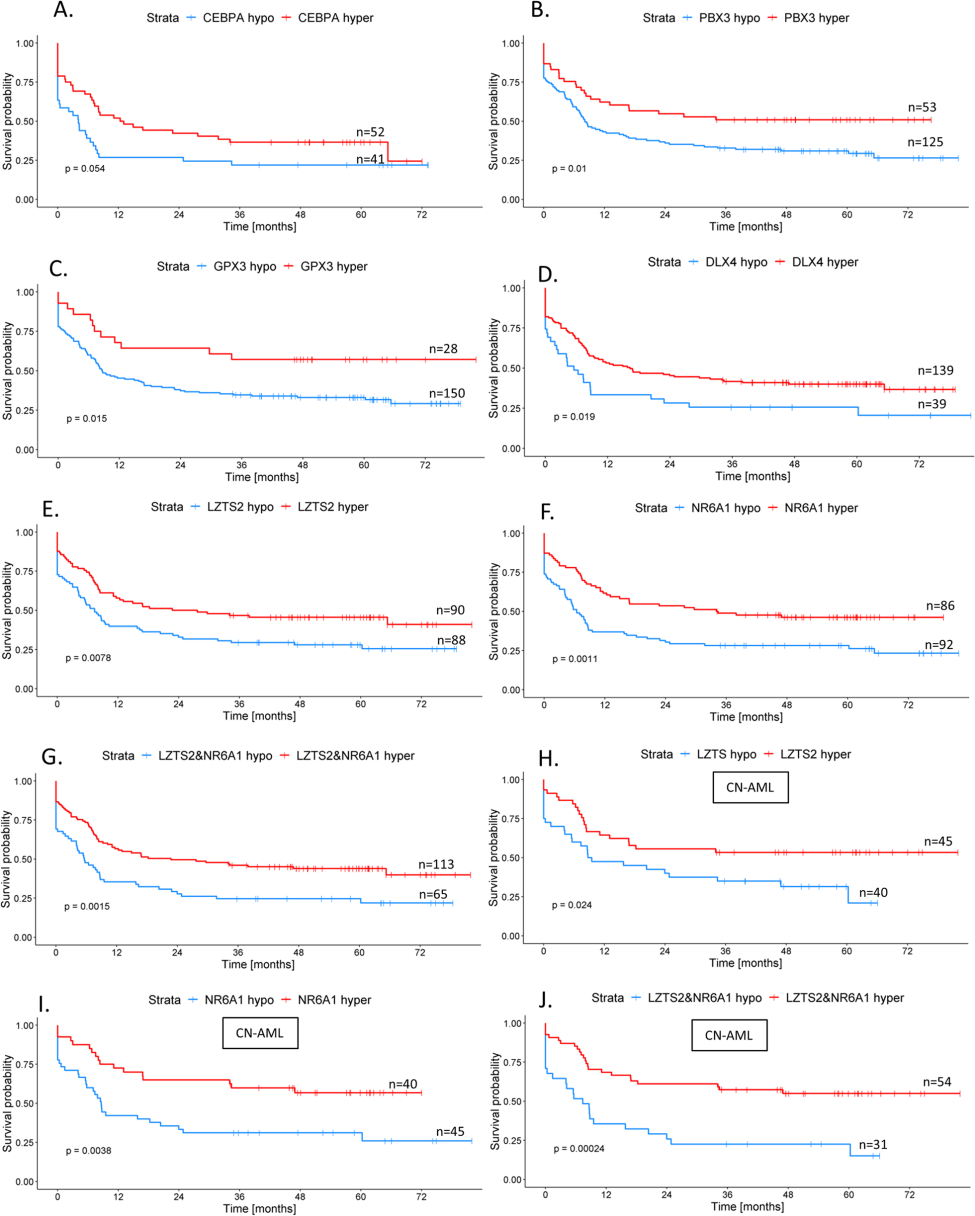
Kaplan–Meier (KM) curves for event-free survival (EFS): **A** *CEBPA* methylation KM curves in AML subgroup excluding favorable cytogenetics and without *CEBPA* and *NPM1* mutations (*n* = 83). **B** *PBX3* methylation KM curves in the whole non-M3 AML cohort (*n* = 178). **C** *GPX3* methylation KM curves in the whole non-M3 AML cohort (*n* = 178). **D** *DLX4* methylation KM curves in the whole non-M3 AML cohort (*n* = 178). **E** *LZTS2* methylation KM curves in the whole non-M3 AML cohort (*n* = 178). **F** *NR6A1* methylation KM curves in the whole non-M3 AML cohort (*n* = 178). **G** *LZTS2*&*NR6A1* methylation KM curves in the whole non-M3 AML cohort (*n* = 178). **H** *LZTS2* methylation KM curves in the CN-AML subgroup (*n* = 85). **I** *NR6A1* methylation KM curves in the CN-AML subgroup (*n* = 85). **J** *LZTS2*&*NR6A1* methylation KM curves in the CN-AML subgroup (*n* = 85). CN-AML = cytogenetically normal AML, hypo = hypomethylated, hyper = hypermethylated, Strata—stratified by a variable

**Fig. 3:**
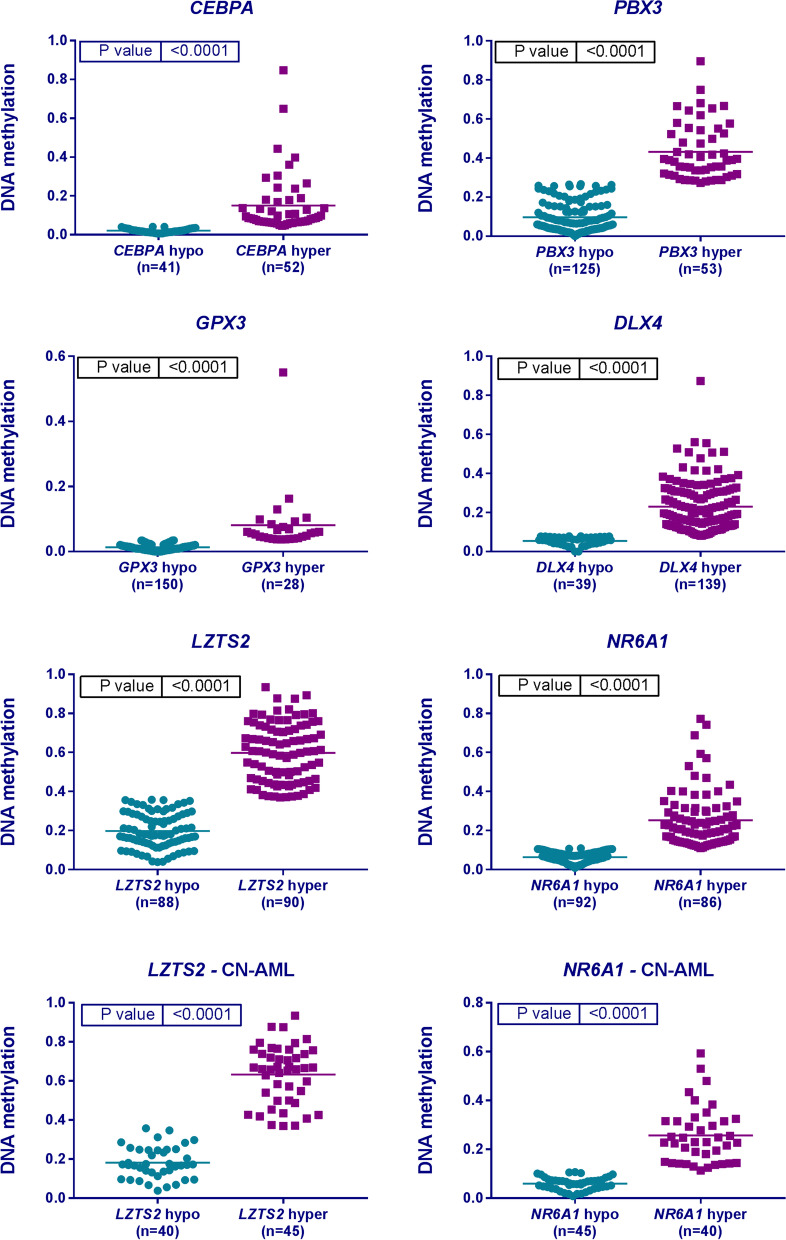
Comparison of mean DNA methylation values in successfully validated genes between hypo- and hypermethylated subgroups of AML. CN-AML = cytogenetically normal AML, hypo = hypomethylated, hyper = hypermethylated

Our validation study confirmed association of DNA methylation status and prognosis for four genes: *CEBPA* [13], *PBX3* [10], *UZTS2* [16], and *NR6A1* [16]. A summary of the results is presented in Table 2. Surprisingly, for two studies [19, 20], we found the exact opposite effect of DNA methylation on prognosis than originally reported—higher *GPX3* and *DLX4* methylation—was linked to a better outcome according to our data. Kaplan–Meier curves for OS and EFS for all six significant genes are shown in Figs.  1 and 2, respectively. In four additional studies [8, 9, 15, 21], only the results from log-rank test displayed statistical significance that was lost in the subsequent multivariate testing (Table 2). These results were not considered as sufficiently conclusive for classifying them as validated. The mean DNA methylation values in hypo- versus hypermethylated subgroups for each of the significant genes are depicted in Fig. 3.

**Table 2:** DNA methylation validation results

Publication	Gene/region tested	Methylation threshold	Mean methylation levels in healthy donors (*n* = 11)	Logrank test		Multivariate Cox analysis^a^ of results significant in Kaplan–Meier analysis	
				*p*-value for OS	*p*-value for EFS	*p*-value for OS	*p*-value for EFS
Lin et al*.* [13]	*CEBPA* distal promoter	4.4%—Cutoff Finder [35]	6%	**0.005**^b^**/0.3**^b^	**0.05**^b^**/0.6**^c^	**0.02**^b^**/-**	**0.03**^b^**/-**
Hájková et al*.* [8]	*CDKN2B, ESR1, MYOD1, CALCA, SOCS1, CDH1*	cumulative methylation value^d^ ≥ 6 (median cumulative value)	*CDKN2B*—3%, *ESR1*—4%, *MYOD1*—5%, *CALCA*—16%, *SOCS1*—0.4%, *CDH1*—7%	0.10^f^	0.60^f^	–	–
		number of hypermethylated genes^e^ ≥ 4 (median number of hypermethylated genes)		**0.04**^f^	0.10^f^	0.19	–
	*SOCS1* promoter	1% (AML median)		0.20^f^	0.20^f^	–	–
Treppendahl et al*.* [18]	*VTRNA2-1* promoter	10%	38%	0.90	0.50	–	–
		38%		0.70	0.40	–	–
Hájková et al*.* [10]	*PBX3* (*TAF1* binding site)	27% (mean healthy donors)	27%	**0.01**	**0.01**	0.08	**0.01**
Jost et al*.* [11]	1 CpG in *DNMT3A* promoter	10% (AML mean)	1%	1/0.60^g^	1/0.60^g^	–	–
	whole DMR		1%	0.80/0.905^g^	0.80/0.70^g^	–	–
Marcucci et al*.* [15]	*CD34, RHOC, SCRN1, F2RL1, FAM92A1, MIR155HG, VWA8*	10%/10.6%^h^ (AML median of average methylation for all genes)	*CD34*—6%, *RHOC*—14%, *SCRN1*—6%, *F2RL1*—5%, *FAM92A1*—11%, *MIR155HG*—10%, *VWA8*—9%	0.08**/**0.3^h^	**0.01/**0.4^h^	–	0.29/-
		13.7/16.95^h^ (median of weighted summary score^i^)		**0.02/**0.2^h^	**0.01/**0.7^h^	0.8/-	0.9/-
		≥ 6 genes have higher methylation than median in AML		0.1**/**0.2^h^	0.2**/**0.5^h^	–	–
Božić et al*.* [21]	1 CpG in *C1R* 5'UTR region	19% (AML median)	22%	0.30	0.06	–	–
		27% (AML median in the original study)		0.30	0.1		
		40%—Cutoff Finder [35]		**0.02**	**0.03**	0.3	0.1
Zhou et al*.* [19]	*GPX3*	3.6% (mean of healthy donors** + **SD**)**	2%	**0.04**	**0.01**	**0.006**	** < 0.0001**
Zhou et al*.* [20]	*DLX4*	8%—Cutoff Finder [35]	11%	**0.02**	**0.02**	**0.03**	**0.03**
Guo et al*.* [9]	*SFRP1* promoter	12% (AML mean)/10%—Cutoff Finder [35]	*SFRP1*—4% *SFRP2*—3%	0.07/**0.02**	**0.02**/**0.02**	-/0.21	0.06/0.06
	*SFRP2* promoter	6% (AML mean)/5%—Cutoff Finder [35]		0.3/0.3	0.5/0.8	–/–	–/–
	*SFRP1, SFRP2*	9% (AML mean)/8.5%—Cutoff Finder [35]		0.1/**0.05**	0.06/**0.04**	-/0.44	-/0.08
Li et al*.* [12]	*NKD2* promoter	6%—Cutoff Finder [35]	3%	0.5	0.2	–	–
		11.5%—Cutoff Finder [35], CN-AML		0.09	0.1	–	–
Liu et al*.* [14]	*RASSF1A* promoter	0.4% (AML mean)	1%	0.4	0.4	–	–
Qu et al*.* [16]	*LZTS2*	37% (AML median)	*LZTS2*—57% *NR6A1*—16%	**0.02/0.01**^h^	**0.008/0.02**^h^	**0.05/0.01**^h^	**0.0003/0.01**^h^
	*NR6A1*	11% (AML median)		**0.001/0.002**^h^	**0.001/0.004**^h^	**0.004/0.0002**^h^	**0.0003/0.0005**^h^
	*LTZS2, NR6A1*	methylation < median methylation level in both genes		**0.001/0.0001**^h^	**0.001/0.0002**^h^	**0.01/ < 0.0001**^h^	** < 0.0001/ < 0.0001**^h^
Šestáková et al*.* [17]	2 CpGs in *GZMB* associated IGR	45% (AML mean) at both/one/none of the two CpGs	21%	0.10	0.10	–	–

CN-AML, cytogenetically normal AML; DMR, differentially methylated region; IGR, intergenic region; SD, standard deviation

^a^Multivariate analysis with following covariates: age, leukocyte count, cytogenetics (Grimwade, 2010), transplantation in the first complete remission, *FLT3*-ITD, *NPM1*^mut^

^b^Excluded patients with favorable cytogenetic profile, *NPM1*^mut^ a *CEBPA*^mut^

^c^CN-AML patients without *NPM1*^mut^, *CEBPA*^mut^

^d^Cumulative methylation value = (1·number of hypermethylated genes with methylation < 15%) + (2·number of hypermethylated genes with methylation 15–50%) + (3·number of hypermethylated genes with methylation > 50%)

^e^Hypermethylated = methylation higher than maximum methylation detected in healthy donors

^f^Excluded patients with favorable cytogenetic profile

^g^*DNMT3A*^mut^ patients excluded

^h^cytogenetically normal (CN) AML

^i^weighted summary score of dichotomized methylation values calculated according to Marcucci et al*.* [15]

## Discussion

Despite a large number of studies addressing the importance of DNA methylation changes for AML prognosis, these aberrations are still not considered for risk stratification, although many promising results have been already reported. The lack of independent validation studies is probably the main obstacle that does not allow the implementation of epigenetic markers alongside the well-established genetic ones. Most of the publications present just more new potential epigenetic biomarkers, making the actual role of DNA methylation harder to grasp and interpret for clinical purposes. With the aim to verify the prognostic role of specific and already described DNA methylation changes in AML, we designed our custom NGS-based DNA methylation panel that covers 27 genes (Additional file 1: Table S1) taken from 14 studies published between years 2011 and 2019. The reported prognostic significance was verified for three studies [10, 13, 16]. These three studies do not share any apparent features such as size of test cohort, presence of a validation cohort, methodology, or biological material utilized for the DNA methylation assessment (see Table 1). We briefly summarize and discuss the genes with a confirmed role of DNA methylation in AML prognosis. *CEBPA* is a well-known gene involved in AML pathogenesis. Double *CEBPA* mutations have been connected to better OS and EFS [4]. Con cordantly, hypermethylation of distal *CEBPA* promoter was reported as a favorable prognostic biomarker, which we proved in AML subgroup excluding favorable cytogenetics and without *CEBPA* and *NPM1* mutations, but not in CN-AML without *CEBPA* and *NPM1* mutations as also originally described by Lin et al*.* [13]. *PBX3* has been identified as an oncogene in AML that transcriptionally regulates *HOXA* genes and promotes cell proliferation and resistance to chemotherapeutical agents [22]. Hajkova et al*.* [10] reported *PBX3* overexpression associated with a higher incidence of relapses. They also showed a clear correlation between *PBX3* overexpression and hypomethylation. In line with this, we detected *PBX3* hypomethylation as an independent negative prognostic factor for EFS. Qu et al*.* [16] identified higher methylation in CpG island (CGI) shores of *LZTS2* and *NR6A1* genes as a predictor of better prognosis in CN-AML. Interestingly, we confirmed the predictive role of *LZTS2* and *NR6A1* hypermethylation not only in CN-AML, but in the whole non-M3 diagnostic AML cohort as well. The strongest link between DNA methylation and prognosis was observed if the concurrent hypermethylation of both genes was present. Validation of the works of Zhou et al*.* [19, 20] produced contradictory results to the original studies. Unlike them, we observed a clear association between higher *GPX3*/*DLX4* promoter methylation and better survival. This discrepancy is hard to explain because even usage of different methodology (qMSP versus NGS) or biological material (BM versus PB) would not completely reverse the impact of particular gene’s hypermethylation. The recent GPX3 review described its dichotomous role in different cancer types; it can act as either an oncogene or a tumor suppressor [23]. Tumors with high *GPX3* expression have an increased resistance to chemotherapy due to the GPX3 involvement in the antioxidant enzyme system [24]. This would support our findings about *GPX3* hypermethylation (and thus probable downregulation) and favorable outcome in AML cohort treated by standard 3 + 7 induction regimen. As for *DLX4*, its overexpression was described in numerous tumor types (including AML) in association with tumor progression and/or invasion [25–28]. This again supports the link between *DLX4* hypermethylation and better AML prognosis. 

Noticeably, all verified prognostic DNA methylation changes have one thing in common: higher methylation equals better prognosis. Six out of fourteen studies subjected to the validation reported higher methylation/lower expression and superior outcome. From these six studies, three were verified by both log-rank and multivariate Cox regression analysis [10, 13, 16] and three showed significance by log-rank test [8, 15, 21]. On the other hand, from eight studies describing the relationship between higher methylation and poor prognosis, only one displayed significance by log-rank test [9], none was verified by multivariate Cox regression analysis, and for two studies the opposite relation between higher methylation and prognosis was revealed [19, 20]. Altogether, it seems that higher methylation has predominant influence on prognosis in AML. However, the exact location of differential methylation and what specific genes are affected are probably the key elements determining the direction of how DNA methylation influences patients’ outcome.

In three studies, the indirect relation of DNA methylation (through its association with gene expression) and prognosis was reported [10, 12, 15]. From these, only one study was validated [10]. Technically speaking, we cannot exclude the role of gene expression deregulation in patients’ outcome in the remaining two studies [12, 15], because in our study design we did not examine the impact of gene expression on AML prognosis.

Another important aspect to discuss is the usage of PB versus BM for DNA methylation assessment. Our AML cohort consists of PB samples only, whereas PB alone was a starting material in 3/14 studies that underwent validation. Some articles have already dealt with the comparison of DNA methylation results obtained from PB versus BM, and they reported their interchangeability for these purposes [8, 10, 16]. In line with this, the result of DNA methylation validation was not determined by the biological material used. In fact, genes with validated role of their methylation status in AML prognosis were all revealed in studies using either BM alone [13, 19, 20] or studies using a combination of PB and BM [10, 16]. PB is a starting material that is easily accessible to the majority of laboratories and it is not as burdensome for patients as BM aspirates.

In practical terms, implementation of a new biomarker represented by a single gene/region is always more feasible than that of a complex methylation pattern. The low number of genes for which we confirmed the prognostic impact with our NGS-based approach highlights the importanc e of such validation and a need for a consistent and easily reproducible approach to assess the impact of various changes in DNA methylation on AML prognosis.

## Conclusions

We showed that validation of previously published prognostically significant DNA methylation changes is essential to confirm their relevance for patients’ stratification. Out of 27 genes, a statistically significant correlation between DNA methylation status and prognosis was proved for six of them: *CEBPA*, *PBX3*, *LZTS2*, *NR6A1*, *GPX3*, and *DLX4*. We propose that further independent validation studies may build upon our results, because only markers properly verified by several independent studies can be considered for AML prognosis refinement in clinical practice.

## Methods

### Patients

We examined 178 adult AML patients: 128 patients from the Institute of Hematology and Blood Transfusion (Prague, Czech Republic) and 50 patients from the University Hospital Brno (Brno, Czech Republic). All patients were diagnosed with AML between 2013 and 2016 and were treated with curative intent starting with 3 + 7 induction regimen [29]. The clinical and basic molecular characteristics used for statistical analysis are stated in Additional file 1: Table S2. Healthy donors (*n* = 11) were also analyzed. The study was approved by the Ethics committees of both participating institutions and all patients provided their full consent. The research conforms to The Code of Ethics of the World Medical Assoc iation.

### Targeted bisulfite sequencing

Sequencing libraries consisted of 16–18 samples and were prepared according to the SeqCap Epi protocol (Roche, Basel, Switzerland) with KAPA HyperPrep Kit (Roche). Diagnostic whole-blood DNA from AML patients (800–1200 ng) was first mixed with the Bisulfite-conversion Control (unmethylated DNA from phage lambda) provided in the SeqCap Epi Accessory kit (Roche) and then fragmented either via E220 Focused ultrasonicator (Covaris, Woburn, MA, USA) or Bioruptor Pico instrument (Diagenode, Liège, Belgium) to get an average size of 200 bp. EZ DNA Methylation Lightning Kit (Zymo Research, Irvine, CA, USA) was used for the bisulfite conversion. Pooled samples from each library were hybridized for about 68 h with a custom set of probes (made by Roche Company). The final concentration of the libraries was measured using KAPA Library Quantification Kit (Roche), and the average size of the libraries’ fragments was assessed on 4200 TapeStation System (Agilent Technologies, Santa Clara, CA, USA). Libraries were sequenced on a MiSeq instrument (Illumina, San Diego, CA, USA) using the MiSeq Reagent Kit v2 (300-cycles) (Illumina).

### Sequencing data analysis

FastQC (version 0.11.8) [30] and MultiQC (version 1.7) [31] software was used to check the quality of fastq files. Reads were then trimmed and filtered using Cutadapt (version 2.4) [32] and the quality of reads was checked again. Filtered data were mapped with software Segemehl (version 0.3.4) [33] to human genome version GRCh37/hg19 with added sequence of Enterobacteria phage lambda NC_001416.1. Mapping statistics were assessed and we checked that more than 80% of reads were mapped for each sample. Bam files containing mapped reads were sorted and indexed by Samtools software (version 1.10). Subsequently, we used Haarz tool (version 0.3.4) [33] with enabled "callmethyl" option to select methylated positions and create vcf files that were further processed in R software. Positions that corresponded to the lambda phage sequence were separated and used to check that the bisulfite conversion ratio was > 99% for each sample. Remaining positions were filtered and only CpG positions were left in the data. Finally, we selected regions corresponding to loci published in the original articles results and the average methylation across the regions was assessed. The list of selected regions is provided in Additional file 1: Table S1. Raw sequencing data are available at the Gene Expression Omnibus repository (accession number GSE165435).

### Statistical analyses and definitions

For the statistical analyses, R software (version 4.0.0) was used. Surviving patients were censored to the April 6, 2020. Overall survival (OS) was established as time from diagnosis until death of any cause. Event-free survival (EFS) was established as time from the first complete remission until death or hematological relapse. Multivariate Cox regression analysis was computed with following covariates: age, leukocyte count, cytogenetics [34], transplantation in the first complete remission, presence of *FLT3*-ITD and *NPM1* mutations. For five studies (see Table 2), Cutoff Finder [35] was utilized to determine the optimal DNA methylation threshold. We used the same DNA methylation threshold as originally published or it was set up in the most similar and meaningful way. We also adapted the selection of AML patients because some studies detected a prognostic effect of DNA methylation only in a specific subset of AML such as cytogenetically normal (CN) AML. To properly evaluate the prognostic significance of the studied regions, we performed Kaplan–Meier analysis with log-rank test. Subsequently, we assessed the effect of DNA methylation levels on overall (OS) and event-free survival (EFS) using multivariate Cox regression for those loci significantly affecting OS or EFS in Kaplan–Meier analysis. *p*-value ≤ 0.05 was considered as statistically significant.

## Supplementary Information

**Additional file 1.** List of analyzed regions (positions according to hg19 assembly).

## Data Availability

Raw DNA methylation sequencing data are deposited into GEO repository with the accession number GSE165435 (https://www.ncbi.nlm.nih.gov/geo/query/acc.cgi?acc=GSE165435). The other data supporting the findings of the present study are included in this published article [and its Additional files].
